# Thyroid-Stimulating Hormone (TSH)-Secreting Pituitary Macroadenoma Presenting as Biochemical Hyperthyroidism Without Clinical Symptoms: A Case Report

**DOI:** 10.7759/cureus.99843

**Published:** 2025-12-22

**Authors:** Pouyan Famini, Kasra Navabi

**Affiliations:** 1 Medicine, University of California Los Angeles David Geffen School of Medicine, Los Angeles, USA; 2 Medicine/Endocrinology, University of California Los Angeles David Geffen School of Medicine, Los Angeles, USA

**Keywords:** alpha subunit, hyperthyroidism, magnetic resonance imaging and pituitary adenoma, pituitary tumour, tsh-oma

## Abstract

Thyroid-stimulating hormone (TSH)-secreting pituitary adenomas (TSH-omas) are a rare cause of hyperthyroidism. We present a case of a 46-year-old male who was incidentally diagnosed with hyperthyroidism during an annual physical examination, with elevated TSH and free T4 consistent with central hyperthyroidism. Diagnosis of TSH-oma requires a combination of biochemical testing, including measurement of the alpha subunit and the alpha-subunit/TSH molar ratio, pituitary imaging, and differential diagnosis to exclude resistance to thyroid hormone (RTH). Further evaluation for the patient confirmed an elevated alpha subunit and a ratio supportive of TSH-oma. A pituitary MRI revealed a pituitary macroadenoma. Surgery is the primary treatment modality, and the patient underwent successful endoscopic endonasal removal of the pituitary macroadenoma. Postoperative biochemical testing six weeks after surgery showed normalization of thyroid function.

## Introduction

Thyrotropin (TSH)-secreting pituitary adenomas (TSH-omas) represent a rare and often diagnostically challenging cause of hyperthyroidism, accounting for approximately 0.5% to 2% of all pituitary adenomas [1-3]. These tumors are characterized by inappropriate TSH secretion in the presence of elevated thyroid hormone levels, a pattern that contrasts with the suppressed TSH observed in primary hyperthyroidism. Challenges in early diagnosis, related to their low prevalence and subtle clinical presentation, often result in delays, with TSH-omas frequently identified as macroadenomas (>1 cm) [1,2]. The diagnosis requires a comprehensive evaluation, including biochemical confirmation of inappropriate TSH secretion, measurement of serum alpha subunit, and pituitary imaging [4-6]. We present the case of a 46-year-old male who was incidentally found to have central hyperthyroidism during routine screening, leading to the identification and successful surgical resection of a TSH-secreting pituitary macroadenoma. This case highlights the importance of including TSH-omas in the differential diagnosis for patients with discordant thyroid function tests, even in the absence of classic hyperthyroid symptoms.

## Case presentation

A 46-year-old male with a history of hypertension was incidentally found to be hyperthyroid during routine laboratory evaluation with his primary care physician in July 2024. Laboratory evaluation at that time revealed abnormal thyroid function tests, including TSH 6.5 mIU/L (nl 0.3-4.7), Free T4 2.20 ng/dL (nl 0.80-1.70), and Free T3 574 pg/dL (nl 222-383) (Table 1). He had no prior history of thyroid disease, and a TSH measured five months earlier was normal at 3.4 mIU/L, though Free T4 had not been assessed. 

**Table 1:** Thyroid function tests over time demonstrating central hyperthyroidism in a patient with TSH-secreting pituitary adenoma *Laboratory assessment approximately six weeks postoperatively. TSH: Thyroid-stimulating hormone

Date	TSH (0.3–4.7 mIU/L)	Free T4 (0.80–1.70 ng/dL)	Free T3 (222–383 pg/dL) / Total T3 (85–185 ng/dL)	Interpretation
Feb 2024	3.4	–	–	Normal thyroid function
Jul 2024	6.5 ↑	2.20 ↑	Free T3: 574 ↑	Elevated TSH with elevated thyroid hormones, consistent with central hyperthyroidism
Oct 2024	5.7 ↑	2.80 ↑	Total T3: 233 ↑	Persistent central hyperthyroidism confirmed
Jan 2025*	2.1	1.30	Total T3: 132	Normalized thyroid function after pituitary surgery

He was referred to Endocrinology in October 2024. He denied hyperthyroid symptoms aside from headaches attributed to poorly controlled hypertension. He reported no visual changes, palpitations, tremors, weight changes, heat intolerance, or mood disturbances. Vital signs and physical examination were normal, including a thyroid exam, with no stigmata of acromegaly. Ophthalmologic evaluation revealed normal visual fields. There was no family history of thyroid or pituitary disease. 

**Figure 1:**
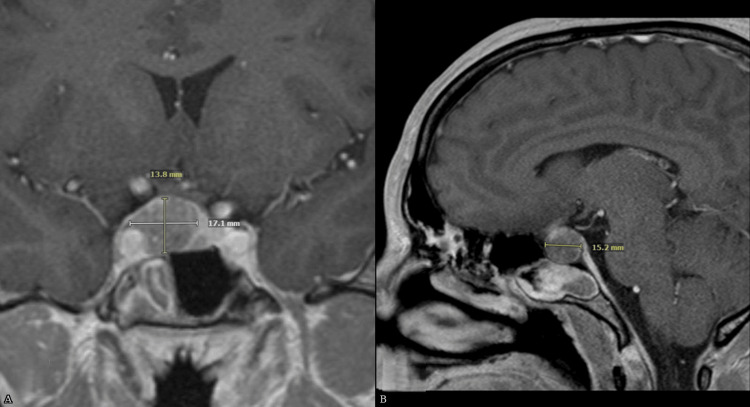
Pituitary magnetic resonance imaging (MRI) demonstrating a thyrotropin (TSH)-secreting pituitary macroadenoma. (A) Coronal T1-weighted post-contrast MRI image showing a sellar mass measuring 17.1 × 13.8 mm centered in the right pituitary with upward displacement of the optic chiasm and prechiasmatic right optic nerve. (B) Sagittal T1-weighted post-contrast MRI image demonstrating superior extension of the macroadenoma measuring 15.2 mm in craniocaudal dimension.

Repeat thyroid function tests in October 2024 showed TSH 5.7 mIU/L, free T4 2.8 ng/dL, and total T3 233 ng/dL (nl 85-185). The alpha subunit was elevated at 2.2 ng/mL (<0.86), and other anterior pituitary hormones, including IGF-1, prolactin, testosterone, and cortisol, were normal. Resistance to thyroid hormone (RTH) was considered but excluded based on the absence of family history, lack of clinical features, and confirmatory alpha-subunit measurement. Assay interference was excluded by repeat testing on different immunoassay platforms, including free T4 measurement by equilibrium dialysis, supporting the diagnosis of a TSH-secreting pituitary adenoma. Pituitary MRI demonstrated an expansive sellar mass centered on the right pituitary gland measuring 17 × 14 × 15 mm, with small cystic areas, upward displacement of the optic chiasm and prechiasmatic right optic nerve, and broad contact with the right cavernous internal carotid artery. The pituitary gland and infundibulum were shifted leftward. Pituitary magnetic resonance imaging findings are illustrated in Figure 1, demonstrating the sellar mass consistent with a thyrotropin-secreting pituitary macroadenoma.

The patient underwent an uncomplicated endoscopic endonasal transsphenoidal resection of the pituitary macroadenoma in December 2024. Surgical pathology showed a thyrotroph-type pituitary neuroendocrine tumor with up to 70% of tumor cells positive for TSH and 50% staining for growth hormone. Outpatient laboratory evaluation six weeks postoperatively revealed normalization of thyroid function as well as normal IGF-1, prolactin, testosterone, and cortisol. The progression of thyroid function tests over time, including postoperative normalization, is summarized in Table 1.

## Discussion

Thyroid-stimulating hormone (TSH)-secreting pituitary adenoma is a rare case that is characterized by high or inappropriately normal thyrotropin levels along with an increase in thyroid hormones that lead, in most patients, to signs and symptoms similar to those of patients with primary hyperthyroidism. These tumors account for approximately 0.5 to 2% of all pituitary adenomas [1-3] and are an even rarer cause of hyperthyroidism [6]. The use of ultrasensitive TSH assays has allowed for a clear distinction between primary hyperthyroidism and central hyperthyroidism, as prior radioimmunoassays did not always differentiate between suppressed and non-suppressed circulating TSH concentrations. Furthermore, thyroid function tests are now being assessed more frequently than they were in the past, leading to increased cases of diagnosis of TSH-omas, with many cases being diagnosed at an earlier stage. Despite these improvements, the majority of TSH-omas are still diagnosed as macroadenomas (>1 cm) [2,5].

Furthermore, many TSH-omas classified as macroadenomas may be asymptomatic. In a historical series of 25 patients followed at the National Institutes of Health over 14 years (1982-1996), 3 of 23 patients with macroadenomas, about 13%, had no hyperthyroid symptoms at presentation, and in a more recent single-center study of 152 patients with TSH-secreting macroadenomas followed from 2001 to 2022, approximately 24% of patients were clinically asymptomatic at diagnosis [7,8]. Patients with macroadenomas may also have symptoms of invasion and compression from the mass to the surrounding area, including headaches and visual disturbances. Furthermore, some patients may have clinical features of concomitant hypersecretion of other pituitary hormones such as acromegaly or galactorrhea/amenorrhea, as about one-quarter of TSH-omas have been found to cosecrete other pituitary hormones, most commonly growth hormone and prolactin [4]. Moreover, pituitary tumors that demonstrate immunohistochemical expression of one or more anterior pituitary hormones but lack clinically significant hormone secretion are designated as silent pituitary adenomas [3]. However, the absence of clinical symptoms can often make it difficult to distinguish TSH-oma from other conditions. Interpretation of thyroid function test patterns and diagnostic next steps are summarized in Table 2. 

**Table 2:** Interpretation of TSH and free T4 patterns and next steps TSH: Thyroid-stimulating hormone

TSH	Free T4	Possible Diagnosis	Next Steps / Considerations
High / Inappropriately normal	High	Central hyperthyroidism (TSH-oma, thyroid hormone resistance)	Measure alpha subunit, repeat thyroid function on alternate assay, perform pituitary MRI, and consider genetic testing for RTH if indicated
Low	High	Primary hyperthyroidism (Graves disease, toxic adenoma, toxic multinodular goiter)	Evaluate thyroid autoantibodies (TRAb), thyroid uptake scan, and treat underlying thyroid disease
Low	Low	Secondary hypothyroidism (pituitary failure)	Evaluate other pituitary hormones, pituitary MRI, and consider replacement therapy
Normal	High	Early or subclinical hyperthyroidism, assay interference possible	Repeat tests, consider clinical context, and evaluate for assay interference
Normal	Low	Secondary hypothyroidism (pituitary failure) or non-thyroidal illness (including recovery phase)	Evaluate other pituitary hormones, pituitary MRI, and consider replacement therapy or monitor and treat underlying illness

Thyroid hormone resistance (RTH) results in elevated thyroid hormone levels with non-suppressed TSH, but a lack of clinical thyrotoxicosis, and genetic testing or family history can help confirm the diagnosis [9]. Assay interference, such as heterophile antibodies or abnormal binding proteins, may produce spurious results and can be excluded by repeating tests on alternative assay platforms [10]. The measurement of the alpha subunit, a common component of pituitary glycoprotein hormones including TSH, can help distinguish between a TSH-secreting pituitary adenoma and other causes. An elevated alpha subunit and alpha-subunit/TSH ratio above 1.0 have been shown to have sensitivities of 90% and 90% and specificities of 82% and 73% for TSH-omas, respectively [7]. Imaging modalities used to diagnose TSH-omas include pituitary MRI, which is the most commonly used imaging modality and the study of choice, as well as radiolabeled somatostatin scintigraphy and positron emission tomography (PET) scans [5].

The European Thyroid Association guidelines advocate pituitary adenectomy, usually via the trans-sphenoidal approach, for restoring thyroid function [11]. Pituitary fractionated stereotactic radiotherapy or radiosurgery, and also medical therapy with somatostatin analogs, can be considered in cases of surgical failure or if there are contraindications to surgery. TSH-omas seem to have a low rate of recurrence after successful surgery [12]. Long-term follow-up includes clinical and biochemical evaluation two to three times during the first postoperative year and annually thereafter. This evaluation should include measurement of TSH and circulating free thyroid hormones and, if indicated, assessment of other pituitary hormones. Pituitary imaging is recommended every two to three years, or sooner if there is a rise in TSH and thyroid hormone levels or the emergence of clinical symptoms [11].

## Conclusions

TSH-secreting pituitary adenomas are a rare but clinically important cause of central hyperthyroidism. Accurate diagnosis requires careful biochemical evaluation, including measurement of the alpha subunit and calculation of the alpha-subunit/TSH molar ratio, which, when elevated above 1.0, strongly supports the presence of a TSH-oma. Imaging is crucial to identify the pituitary adenoma, define its size and extent, and distinguish it from other causes of central hyperthyroidism. Transsphenoidal surgical resection remains the treatment of choice, providing high rates of biochemical remission and a low risk of recurrence. Long-term follow-up with periodic biochemical and imaging assessments is essential to detect potential recurrence and evaluate pituitary function over time. This case underscores the importance of considering TSH-omas in patients with inappropriately normal or elevated TSH in the setting of hyperthyroidism, even in the absence of classic clinical symptoms. Early recognition and prompt surgical management can not only achieve rapid biochemical remission but also prevent complications related to tumor growth, mass effect, or co-secretion of other pituitary hormones, ultimately improving patient outcomes.
